# RANKL is a therapeutic target of bone destruction in rheumatoid arthritis

**DOI:** 10.12688/f1000research.17296.1

**Published:** 2019-04-23

**Authors:** Sakae Tanaka

**Affiliations:** 1 Department of Orthopaedic Surgery, Faculty of Medicine, The University of Tokyo, 7-3-1 Hongo, Bunkyo-ku, Tokyo, 113-0033, Japan

**Keywords:** Denosumab, osteoclast, RANKL, rheumatoid arthritis

## Abstract

Although remarkable advances have been made in the treatment of rheumatoid arthritis (RA), novel therapeutic options with different mechanisms of action and fewer side effects have been expected. Recent studies have demonstrated that bone-resorbing osteoclasts are critically involved in the bone destruction associated with RA. Denosumab, a human antibody against receptor activator of nuclear factor-kappa B ligand (RANKL), efficiently suppressed the progression of bone erosion in patients with RA by suppressing osteoclast differentiation and activation in several clinical studies, although it had no effect on inflammation or cartilage destruction. Denosumab, in combination with anti-rheumatic drugs, is considered a pivotal therapeutic option for the prevention of bone destruction in RA.

## Introduction

Rheumatoid arthritis (RA) is an inflammatory disorder of unknown etiology, characterized by chronic inflammation of the synovial joints through autoimmune mechanisms ^1, 2^. Remarkable progress in the treatment of RA has been achieved during the last 20 years. Methotrexate (MTX) has been considered an anchor drug and is used as the first-line therapeutic modality for RA. MTX monotherapy was reported to achieve repair of severely damaged joints (radiographic healing) by suppressing joint inflammation ^3^. The emergence of biological agents like tumor necrosis factor (TNF) inhibitors has had a remarkable impact on therapeutic strategies for RA and greatly improved disease control in patients with RA. In addition, two targeted synthetic disease-modifying anti-rheumatic drugs—tofacitinib and baricitinib—that target Janus kinases have recently been introduced and showed equal efficacy to biologics in the treatment of RA. Despite the excellent effects of these novel therapeutic agents, there are still several limitations in their treatment of RA. First, and most importantly, almost all currently available anti-rheumatic drugs basically suppress the immunological function of patients and thus are inevitably associated with a wide range of immunosuppression-related side effects such as infections. Furthermore, the effects of these drugs are not perfect, and a fair number of patients do not respond well to treatment. Therefore, novel therapeutic options with different mechanisms of action and fewer side effects have long been expected.

## Involvement of osteoclasts in bone destruction associated with rheumatoid arthritis

**Figure 1.**
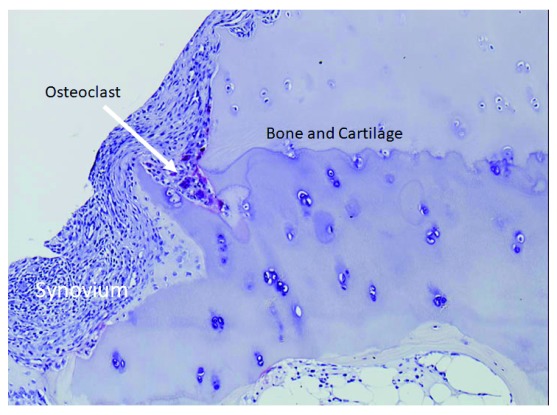
Bone erosion in rheumatoid arthritis. The proliferating synovium stimulates osteoclast differentiation, and many osteoclasts are observed at the synovium–bone interface (arrow).

Osteoclasts are multinucleated giant cells primarily responsible for bone resorption. They originate from hematopoietic stem cells and differentiate from monocyte/macrophage-lineage precursor cells. Previous studies showed that osteoclasts are critically involved in not only physiological bone metabolism but also pathologic bone destruction such as that observed in RA, osteoporosis, and cancer bone metastasis ^4^. In particular, the primary role of osteoclasts in the bone destruction associated with RA has attracted a great deal of attention. Multinucleated giant cells with characteristics of osteoclasts are frequently observed at the interface between the inflammatory synovium and the eroded bone of patients with RA ^5^ ( Figure 1), and Gravallese *et al*. ^6^ reported that these multinucleated cells expressed osteoclast-specific genes like tartrate-resistant acid phosphatase and calcitonin receptor. We previously reported that differentiation to multinucleated osteoclasts was induced when synovial fibroblasts from patients with RA were co-cultured with peripheral blood mononuclear cells in the presence of 1α,25-dihydroxyvitamin D _3_ [1α,25(OH) _2_D _3_] and macrophage colony-stimulating factor (M-CSF) ^7^.

The critical role of osteoclasts in bone destruction associated with RA was confirmed by findings in a rare human case. An inherited disorder characterized by increased bone mineral density (BMD), osteopetrosis arises from a defect in osteoclast differentiation or activation ^8^. We described a patient whose type II autosomal dominant osteopetrosis was diagnosed in his youth and demonstrated markedly reduced osteoclast activity ^9^. The patient coincidentally developed RA but showed a slow progression of bone erosion despite severe inflammation and rapid progression of cartilage destruction ^9^. These results suggest that the normal activity of osteoclasts is required for the bone destruction in RA.

## Regulation of osteoclast development by RANKL–RANK pathways

Receptor activator of nuclear factor-kappa B ligand (RANKL) belongs to the TNF superfamily. RANKL was originally identified as an activated T cell–producing factor that modulates dendritic cell survival ^10, 11^, and many subsequent studies elucidated its essential role in osteoclast biology. RANKL expression is induced in osteoblastic cells and bone marrow stromal cells in response to calciotropic factors such as 1α,25(OH) _2_D _3_ and parathyroid hormone, and combined treatment of hematopoietic cells with M-CSF and a soluble form of RANKL induced osteoclast differentiation *in vitro* ^4, 12^. The receptor for RANKL, RANK, is expressed in monocyte/macrophage-lineage osteoclast precursor cells, and osteoprotegerin (OPG), a member of the TNF receptor superfamily, binds to RANKL and competitively suppresses its binding to RANK. The physiological importance of the RANKL–RANK–OPG axis was confirmed by mouse genetic studies. Targeted disruption of *Rankl* or *Rank*, as well as overexpression of *Opg*, induced osteopetrosis in mice through a defect in osteoclast differentiation, whereas deletion of the *Opg* gene or overexpression of *Rankl* led to a marked reduction in bone mass, mimicking osteoporosis ^13^. Furthermore, several human genetic disorders were shown to be associated with the RANKL–RANK–OPG axis. Patients with *RANKL* or *RANK* gene mutations developed a distinct subgroup of recessive osteopetrosis diseases with very few osteoclasts in their skeletal tissues ^14^. In contrast, familial expansile osteolysis, early-onset Paget’s disease of bone, and expansile skeletal hyperphosphatasia were associated with gene mutations that caused enhancement of RANKL–RANK pathways and thus increased bone resorption ^14^.

## Osteoclasts as a therapeutic target in rheumatoid arthritis

Since osteoclasts are critically involved in the bone destruction associated with RA, osteoclasts are considered a therapeutic target in RA. Bisphosphonates, stable analogues of pyrophosphate, are representative anti-osteoporosis medicines with strong anti-catabolic activity, and several studies have been conducted to investigate their effects in RA. Ralston *et al*. ^15^ analyzed the effect of aminohydroxypropylidene bisphosphonate (APD) in patients with RA. APD treatment did not ameliorate radiographic progression even though biochemical markers of increased bone resorption were significantly suppressed in the APD group. Eggelmeijer *et al*. ^16^ reported that pamidronate treatment increased BMD in patients but did not ameliorate joint damage or disease activity. More recently, the effect of zoledronic acid (ZA) was analyzed in both animal models and patients with RA. Herrak *et al*. ^17^ and Sims *et al*. ^18^ almost simultaneously reported that ZA suppressed bone destruction in human TNF-α transgenic mice and collagen-induced arthritis rats by inhibiting osteoclastic bone resorption, respectively. Moreover, preliminary evidence for a structural benefit of ZA in early-stage RA was reported by Jarrett *et al*. ^19^, whose findings suggest that, when started in the early stage of RA, osteoclast-targeting therapy is effective. However, the clinical evidence is limited.

## RANKL as a therapeutic target in rheumatoid arthritis

Several groups, including ours, reported an increased expression of RANKL in the synovial tissues of patients with RA ^20– 22^. Later, Hashizume *et al*. reported that the expression of RANKL was induced in response to interleukin-6 (IL-6) signaling and that TNF-α, IL-17, or IL-1β stimulated the production of IL-6 in synovial fibroblasts, indicating the involvement of inflammatory cytokines in RANKL production in synovial fibroblasts ^23^. The essential role of RANKL–RANK pathways in arthritic bone destruction was confirmed in a series of animal experiments. OPG treatment ameliorated arthritic bone destruction in adjuvant arthritis rats ^24^ and markedly reduced bone erosion in RANKL-deficient mice with serum transfer-induced arthritis ^25^. In addition, systemic bone loss, as well as local bone erosion, was ameliorated by OPG injection combined with an anti-TNF-α antibody in TNF-α transgenic mice ^26^.

Denosumab is a fully human IgG2a monoclonal antibody that specifically binds to human RANKL and inhibits its interaction with RANK, thereby suppressing bone resorption. In the pivotal Fracture Reduction Evaluation of Denosumab in Osteoporosis Every 6 Months (FREEDOM) study, denosumab treatment for 3 years significantly and continuously increased BMD and reduced the risks of vertebral, non-vertebral, and hip fractures ^27^. Denosumab was effective in treating not only osteoporosis but other pathologic conditions such as bone cancer diseases and giant cell tumor of bone ^28^.

**Figure 2.**
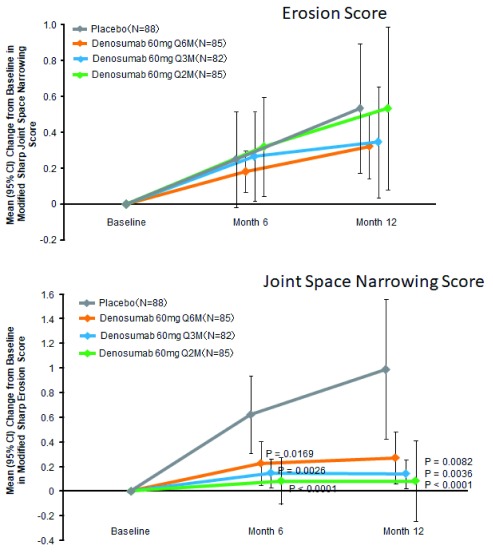
Denosumab significantly suppresses the bone erosion score but has no effect on the joint-space narrowing score determined by modified Sharp scores. ( **a**) Modified Sharp erosion score. ( **b**) Modified Sharp joint-space narrowing score. CI, confidence interval; Q2M, every 2 months; Q3M, every 3 months; Q6M, every 6 months. Modified from a figure in an article by Takeuchi *et al*. ^30^.

The effects of denosumab in patients with RA have been examined in several clinical trials. Cohen *et al*. ^29^ reported that the progression in the erosion score at 6 months on magnetic resonance imaging was lower in the denosumab group compared with the placebo group. In contrast, denosumab had no protective effect on the progression of joint-space narrowing or RA disease activity, probably because it cannot ameliorate the synovial inflammation in RA. The effect of denosumab in Japanese patients with RA was more recently reported ^30^. Patients with RA were randomly assigned to subcutaneous injection of placebo or denosumab 60 mg every 6 months (Q6M), Q3M, or Q2M. Compared with placebo, denosumab at all doses significantly inhibited the progression of bone erosion at 12 months as determined by the modified Sharp erosion score but had no obvious effect on joint-space narrowing ( Figure 2). Notably, no apparent difference in the safety profiles of denosumab and placebo was reported. These results give strong evidence that, in the early stage of RA, denosumab can prevent the progression of bone erosion but has little or no effect on cartilage deterioration or disease activity. Moreover, Hasegawa *et al*. ^31^ reported that, compared with biological agent treatment alone, the concurrent use of denosumab with biological agents was more efficacious in inhibiting structural damage in RA patients without increasing adverse events. Interestingly, Ebina *et al*. ^32^ reported that, compared with continuing oral bisphosphonates or switching to teriparatide, switching oral bisphosphonates to denosumab significantly reduced radiographic joint destruction at 12 months. Based on these results, denosumab obtained approval for “inhibition of the progression of bone erosion associated with RA” in Japan ^33^.

## Conclusions

**Figure 3.**
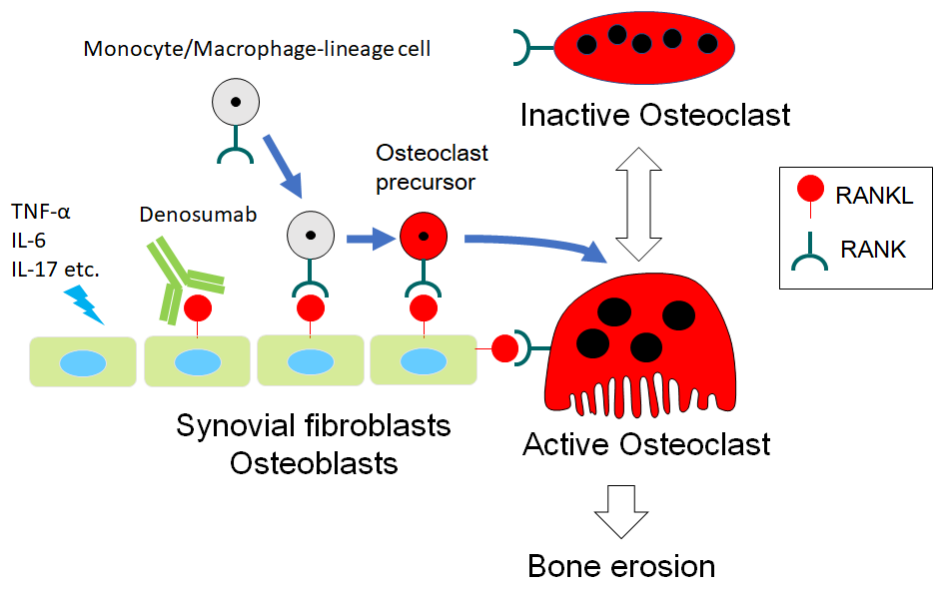
Schematic representation of the mechanism for osteoclast development and denosumab action in rheumatoid arthritis. Pro-inflammatory cytokines such as tumor necrosis factor-alpha (TNF-α), interleukin-6 (IL-6), and IL-17 directly or indirectly induce receptor activator of nuclear factor-kappa B ligand (RANKL) expression in synovial fibroblasts or osteoblasts or both. RANKL stimulates osteoclast differentiation from monocyte/macrophage-lineage precursor cells, leading to bone erosion in rheumatoid arthritis. Denosumab specifically binds to RANKL and suppresses osteoclast differentiation.

We have proposed a novel therapeutic approach to prevent bone destruction in RA by targeting RANKL ^34^ ( Figure 3). Denosumab effectively prevents bone destruction but has no effect on joint inflammation or cartilage destruction in RA. Therefore, denosumab should be used together with other therapeutic agents, like MTX and biologics, for the treatment of RA. However, several clinical questions remain to be addressed. These questions include whether denosumab should be started immediately after diagnosis of RA and whether it can be stopped. The latter question is important because multiple vertebral fractures were reported to be observed after discontinuation of denosumab in patients with osteoporosis ^35^. Further clinical and basic studies are required to address these questions and to establish the appropriate role of denosumab in treatment strategies for RA.
